# Caseous lymphadenitis with cutaneous involvement: A rare presentation of а zoonotic infection

**DOI:** 10.1016/j.jdcr.2026.09.034

**Published:** 2026-09-26

**Authors:** Nikola A. Angelov, Dimitar N. Kurtev, Alexandar I. Kartsakov, Plamena A. Damyanova, Razvigor B. Darlenski

**Affiliations:** a Department of Dermatology and Venereology, MHAT “Saint Panteleimon”, Yambol, Bulgaria; b Department of Dermatology and Venereology, Medical Faculty, Trakia University, Stara Zagora, Bulgaria

**Keywords:** caseous lymphadenitis, chronic skin lesion, Corynebacterium pseudotuberculosis, granulomatous inflammation, zoonosis

## Introduction

Caseous lymphadenitis (CLA) is a rare zoonotic infection caused by *Corynebacterium pseudotuberculosis*, a Gram-positive bacterium that primarily affects livestock, such as sheep and goats.^1^ Human infection is uncommon, with few reported cases since its first description in 1966.^2^^,^^3^ Transmission occurs mainly through direct contact with infected animals or breaches in the skin barrier, although cases associated with unpasteurized dairy products have been described.^3^^,^^4^ In humans, the disease most commonly presents as necrotizing granulomatous lymphadenitis, with rare reports of pulmonary involvement.^1^^,^^5^

### Clinical case

A 61-year-old woman presented with a 1-year history of progressively enlarging skin lesions on the right lower leg. The lesions initially appeared as small, painful subcutaneous nodules that gradually softened centrally and began to drain yellow-to-green purulent material. The clinical course was chronic, with intermittent exacerbations characterized by increased pain, erythema, and swelling, without complete resolution despite multiple courses of outpatient antibiotic therapy with doxycycline and cefuroxime. The patient reported prior contact with livestock, including participation in sheep processing.

On examination, the patient appeared well without fever, weight loss, cough, or other systemic symptoms. Multiple inflammatory nodules and indurated plaques were present on the anteromedial aspect of the right lower leg near the knee. Lesions showed erythematous-to-violaceous discoloration and firm consistency, with areas of central softening, ulceration, and sinus tracts draining purulent material. Crusts, necrotic debris, surrounding erythema, and moderate edema were noted, along with postinflammatory hyperpigmentation and scarring. No clinically enlarged regional lymph nodes were detected (Fig 1, *A*).

**Fig 1:**
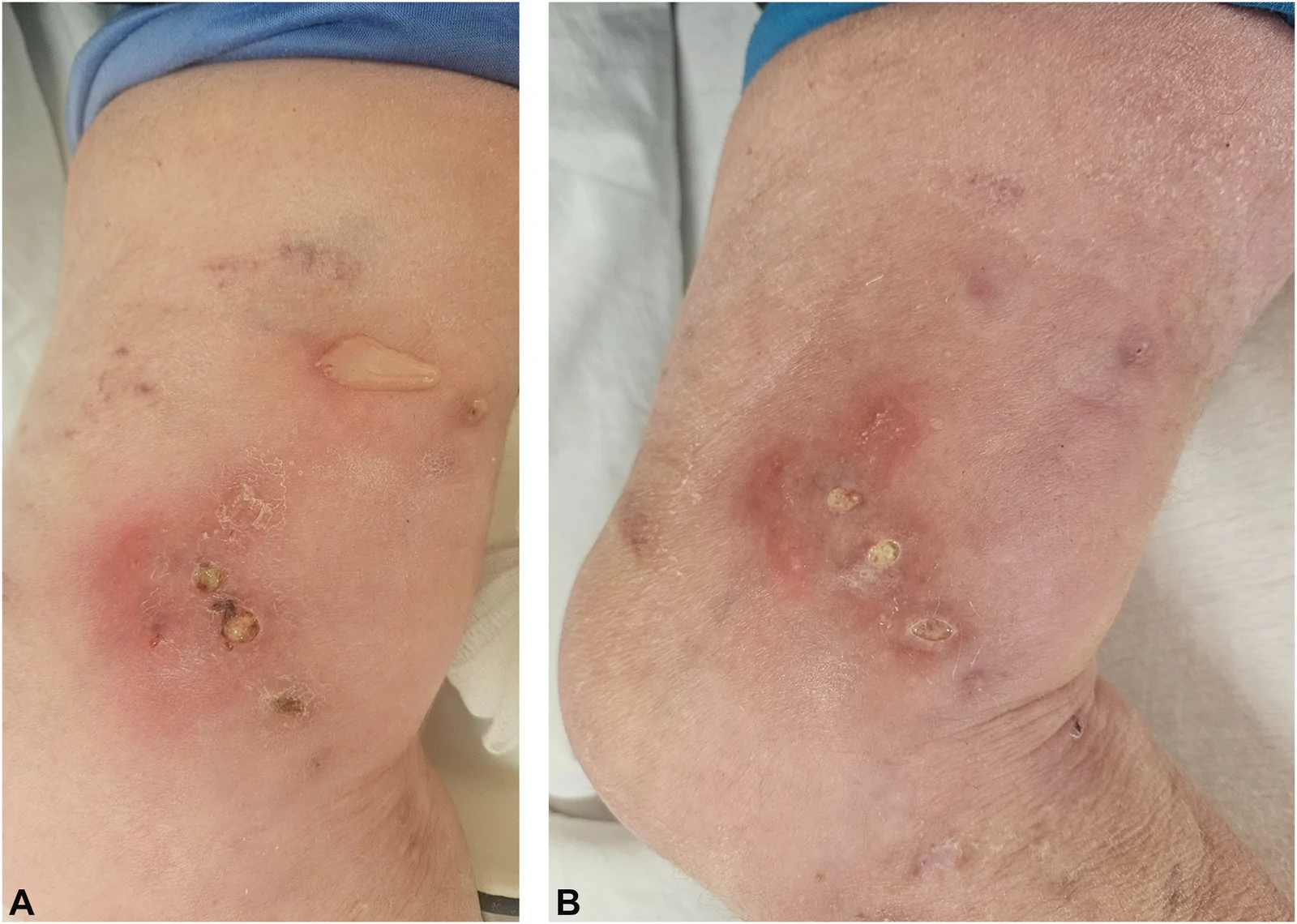
**A,** Multiple inflammatory nodules and indurated infiltrates with erythematous to violaceous discoloration, some showing central necrosis, ulceration, and draining sinus tracts with purulent discharge. **B,** Clinical improvement with reduced inflammation, erythema, and drainage from sinus tracts.

Bacterial culture of purulent material identified *C pseudotuberculosis*, susceptible only to vancomycin and moxifloxacin. Laboratory findings showed neutrophilia, elevated C-reactive protein, and lymphopenia. The interferon-γ release assay (QuantiFERON-TB Gold) was negative.

Ultrasound demonstrated skin thickening with disrupted architecture and multiple fluid-filled cavities containing heterogeneous internal echoes and necrotic debris, as well as draining sinus tracts communicating with the skin surface (Fig 2). Histopathologic examination of the excised skin and subcutaneous tissue demonstrated a chronic suppurative granulomatous inflammatory process (Fig 3, *A*) with multinucleated giant cells (Fig 3, *B*).

**Fig 2:**
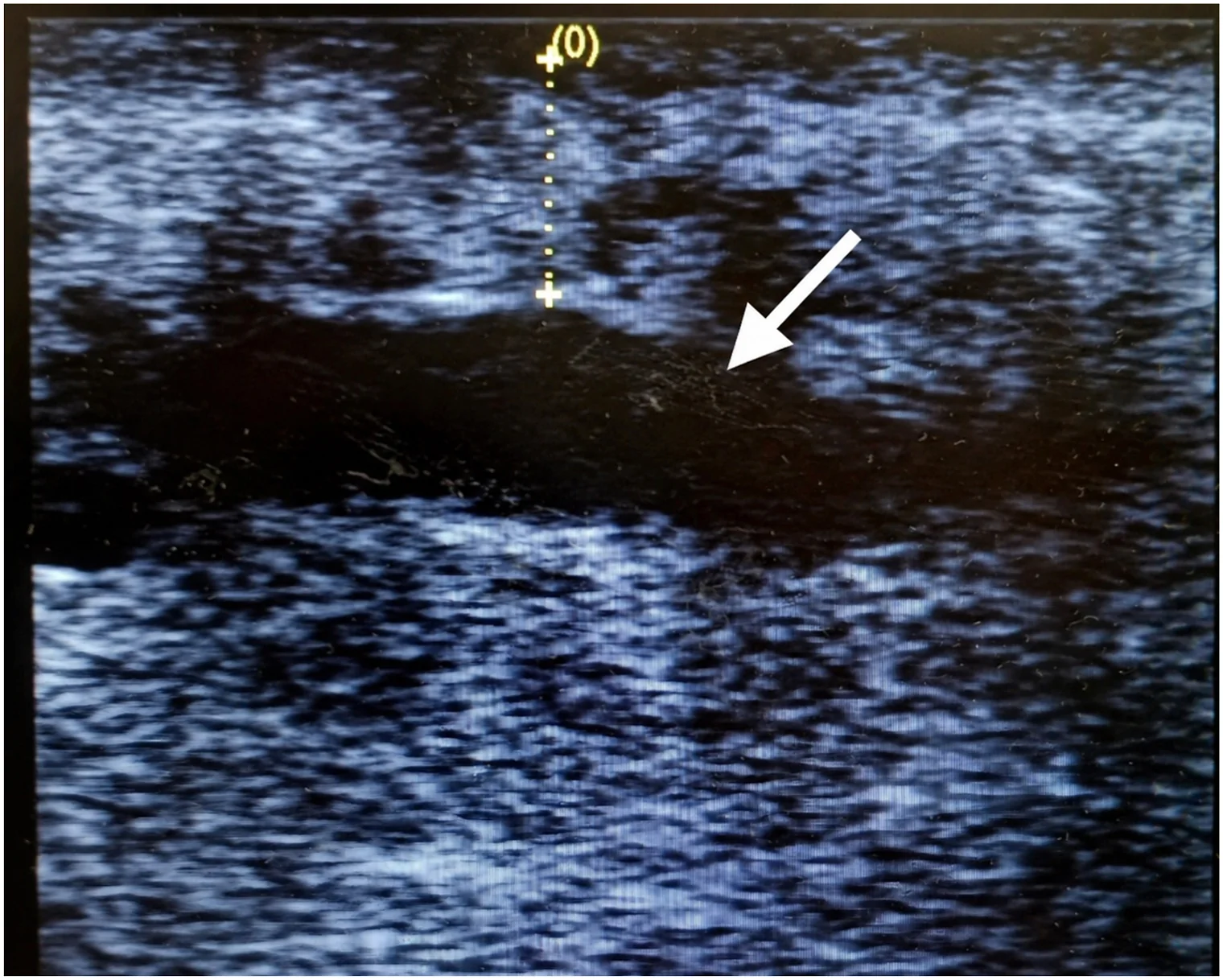
Ultrasound showing subcutaneous fluid-filled cavities with sinus tracts, consistent with a chronic granulomatous process with necrosis.

**Fig 3:**
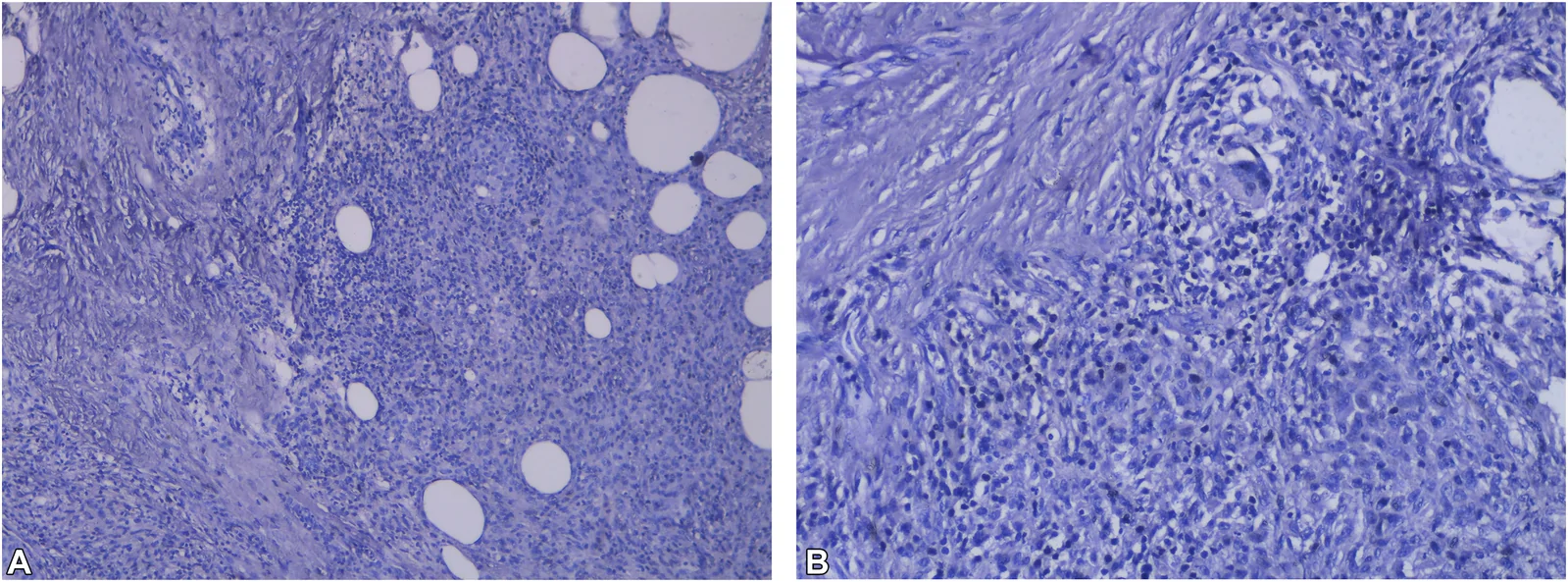
**A,** Histopathological features of chronic suppurative granulomatous inflammation in the excised skin and subcutaneous tissue (hematoxylin–eosin, original magnification × 100). **B,** Multinucleated giant cells within the chronic suppurative granulomatous inflammatory infiltrate (hematoxylin–eosin, original magnification × 200).

The patient was treated with intravenous vancomycin (500 mg twice daily for 7 days), local antiseptic therapy, and drainage of the fluctuant abscesses, resulting in gradual clinical improvement with reduced inflammation, decreased drainage, and partial re-epithelialization (Fig 1, *B*). Given the chronic course, surgical excision of the affected skin and subcutaneous tissue was performed. Postoperatively, the patient received oral moxifloxacin (400 mg once daily for 30 days), with an uneventful recovery and no evidence of recurrence at short-term follow-up.

## Discussion

CLA is a rare zoonotic infection in humans, with limited data available despite its significance in veterinary medicine.^6^ Most of the human cases originated from Australia where the disease is endemic among sheep and goats.^3^ The reported clinical spectrum ranges from granulomatous lymphadenitis to cutaneous and pulmonary involvement (Table I).

**Table I:** Summary of published human cases of *Corynebacterium pseudotuberculosis* infection and comparison with the present case

Author	Year	Country	Clinical presentation	Treatment	Outcome
Lopez et al.^2^	1966	Panama	Granulomatous lymphadenitis	Surgical excision & antibiotics	Recovered
Keslin et al.^5^	1979	USA	Eosinophilic pneumonia	Antibiotics	Recovered
Goldberger et al.^4^	1981	USA	Suppurative granulomatous lymphadenitis	Surgical excision & antibiotics	Recovered
Peel et al (10 case series)^3^	1997	Australia	Granulomatous lymphadenitis	Surgical excision & antibiotics	Protracted or recurrent course
Heggelund et al.^1^	2015	Norway	Pneumonia	Antibiotics	Recovered
Present case	2026	Bulgaria	Chronic cutaneous and subcutaneous lesions	Surgical excision & antibiotics	No recurrence at short-term follow-up
					

The infection in livestock is characterized by chronic abscess formation involving superficial and internal lymph nodes which may rupture through the skin and form chronic draining tracts with purulent discharge, contributing to ongoing risk of transmission to humans.^7^

The differential diagnosis of chronic nodular and draining skin lesions with sinus tracts includes cutaneous tuberculosis, atypical mycobacterial infections, actinomycosis, and deep fungal infections. Microbiological culture of purulent material subsequently confirmed *C pseudotuberculosis* as the causative agent.

Management of CLA in humans may be challenging because of the limited response to antibiotic therapy. In chronic cases, surgical intervention may be required to achieve adequate disease control.

Control measures in livestock include isolation of infected animals and vaccination strategies; however, currently available vaccines provide incomplete protection and variable efficacy.^8^^,^^9^ Appropriate hygiene measures and control of animal-derived products remain important for reducing the risk of human infection.^10^

## Conclusion

CLA should be considered in the differential diagnosis of chronic nodular skin lesions associated with draining sinus tracts particularly in patients with a history of animal exposure. Early recognition and microbiologic confirmation are essential for accurate diagnosis. Combined medical and surgical management may be required in chronic cases to achieve optimal outcomes. This case expands the limited literature on human *C pseudotuberculosis* infection by illustrating a rare predominantly cutaneous presentation.

### Declaration of generative AI and AI-assisted technologies in the writing process

During the preparation of this work, the authors used ChatGPT (OpenAI) for grammar editing. After using this tool, the authors reviewed and edited the content as needed and take full responsibility for the content of the publication.

## Conflicts of interest

None disclosed.
